# miR-383 regulates sheep granular cell proliferation and apoptosis by targeting *Bcl-2*

**DOI:** 10.5194/aab-68-287-2025

**Published:** 2025-05-15

**Authors:** Binglei Zhang, Saiyi Sun, Wanhang Jia, Jiaxin Yang, Xueru Dou, Yang Wang, Yuqin Wang

**Affiliations:** 1 College of Animal Science and Technology, Henan University of Science and Technology, Luoyang 471000, China

## Abstract

During the reproductive process in female mammals, approximately 99 % of the follicles involved in egg development undergo atresia and are not utilised. The primary cause of follicular atresia is granulosa cell (GC) apoptosis. MicroRNAs (miRNAs) play an important regulatory role in follicular atresia. It has been widely confirmed that miR-383 is involved in the regulation of follicular GC proliferation, apoptosis, and steroid hormone secretion; however, its regulatory effect on sheep ovarian follicles remains unknown. In this study, we examined the regulatory role of miR-383 in ovine ovarian GC proliferation and apoptosis. We reveal that miR-383 overexpression induces cell death, inhibits cell proliferation, and causes a G1-phase cell cycle arrest. Based on prediction analysis of its target genes, miR-383 potentially targets the anti-apoptotic gene, *Bcl-2*. A subsequent dual-luciferase reporter assay confirmed this prediction. Overall, the results indicated that miR-383 regulates sheep GC proliferation and apoptosis by targeting *Bcl-2*. This study provides further insights into the regulatory role of miRNAs in ovine follicular development and atresia.

## 1 Introduction

Follicular atresia is a highly orchestrated process that destroys and eliminates follicles and oocytes throughout mammalian ovarian development (Matsuda et al., 2012). Follicular atresia leads to wastage of follicle resources and substantially restricts animal production. Granulosa cells (GCs), the largest cell population in the follicles, are located in different basal layers, including cumulus cells surrounding the oocytes and GCs lining the follicle walls, known as wall GCs (Fan et al., 2019). Wall GCs primarily secrete steroid hormones, whereas cumulus cells mainly form cumulus–oocyte complexes to support and nourish the oocytes. Abnormal apoptosis of GCs is the main cause of follicular atresia (Scott et al., 2018).

MicroRNAs (miRNAs) are small, non-coding RNAs with a length of approximately 22 nucleotides that modulate gene expression by inhibiting or degrading mRNAs (Ha and Kim, 2014). Numerous studies have demonstrated the key role of miRNAs in modulating follicular atresia (Worku et al., 2017). For example, during follicular development in chickens, miR-7 regulates follicular atresia by inhibiting the expression of *KLF4*, which regulates the JAK/STAT3 pathway (Wei et al., 2024). miR-486 considerably inhibits GC activity by targeting *SRSF3*. By downregulating *SRSF3* expression, miR-486 overexpression causes follicular atresia, promotes GC death, and decreases GC growth (Liu et al., 2023). In contrast, miR-129-1-3p attenuates mitochondrial autophagy by downregulating the expression of *MCU*, which protects GCs from oxidative stress-induced autophagy, thereby affecting follicular atresia (Zhu et al., 2023). Among these, miR-383 is a well-known miRNA related to the reproductive system. It exerts regulatory effects on various physiological processes, such as oestrogen production (Yin et al., 2014), spermatogenesis (Tian et al., 2013), lipid metabolism (Wang et al., 2023), and tumour cell migration (Jafarzadeh et al., 2022). miR-383 expression is closely linked to cell apoptosis and proliferation (Xu et al., 2015). For example, miR-383-5p targets cold-inducible RNA-binding protein in human GCs to suppress proliferation and enhance apoptosis (Li et al., 2022b). In the JAK/STAT signalling pathway, miR-383 overexpression inhibits hepatocellular carcinoma growth and promotes apoptosis by suppressing *IL17* expression (Wang et al., 2019). Taken together, these studies suggest that miR-383 can regulate the animal reproductive system by controlling cell apoptosis.

*Bcl-2* is an important apoptotic protein that inhibits apoptosis by binding to *BAX* and blocking cytochrome c release from the mitochondria (Czabotar et al., 2014). A series of cellular apoptotic events are triggered upon the suppression of *Bcl-2* expression. Several miRNAs share the same target gene *Bcl-2*. For example, miR-195 can target *Bcl-2* to regulate nucleus pulposus cell apoptosis, which aggravates intervertebral degeneration (Lin et al., 2021). Moreover, knocking down miR-21 or enhancing *Bcl-2* expression may protect the heart from injury, thereby preventing myocardial cell death (Li et al., 2022a). Recent studies have demonstrated that *Bcl-2* acts as the target mRNA for miR-383. Furthermore, compared with most tissues, miR-383 showed a lower expression, and *Bcl-2* showed a higher expression in gastric carcinoma cells. Further studies indicated that miR-383 can target the 3^′^-UTR of the *Bcl-2* mRNA to inhibit its translation and decrease the survival rate of fluorouracil-treated gastric carcinoma cells (Tao et al., 2021). Based on the abovementioned studies, we examined the regulatory role of miR-383 in sheep GCs and determined the potential relationship between miR-383 and *Bcl-2* in GCs.

## 2 Materials and methods

### 2.1 Experimental animals

Three adult Hu sheep (approximately 1 year old) were raised by the Nation Meat Sheep Experimental Station of China (Luoyang, Henan Province, China). The sheep had the capacity for pregnancy but were not pregnant during this study. Ewes were maintained under the same conditions and had free access to feed and water. After slaughter, the ovaries were collected and placed in a buffered saline solution containing a 1 % penicillin–streptomycin combination at 37 °C .

### 2.2 GC isolation and culture

The ovaries were rinsed thrice with 75 % alcohol and phosphate-buffered saline (PBS) at 37 °C . A sterile blade was used to cut the follicles with diameters of 2–8 mm , after which the follicular fluid was collected. The GCs were isolated via centrifugation at 1500 × g for 10 min . The GCs were cultured in T25 cell culture vials in 5 mL of 10 % DMEM F12 complete medium at 37 °C for 48 h under 5 % ${\mathrm{CO}}_{2}$ conditions.

### 2.3 Immunofluorescence

The GCs were then seeded into a six-well dish (3 × 10^5^ cellsperwell ) and placed in a normoxic incubator at 37 °C for 2 d . Upon reaching 70 % confluency, the cells were fixed in 4 % paraformaldehyde and immersed in 0.2 % Triton X-100 for 10 min , followed by incubation in 2 % bovine serum albumin for 30 min . The anti-follicle-stimulating hormone receptor (FSHR) was added at a dilution of 1:200 and incubated overnight at 4 °C , followed by three washes with PBS. Goat anti-rabbit IgG was added at a dilution of 1:200 and incubated under dark conditions for 60 min . The slides were rinsed with PBS thrice for 5 min each. Then, 4^′^-6-diamidino-2-phenylindole (DAPI) was added, and the cells were incubated for 10 min . After removing the excess DAPI, a drop of glycerine (glycerine : PBS =1:1 ) was applied to the cleaned slide. The slide was covered with a coverslip and sealed around the edges with transparent nail polish before observation using fluorescence microscopy.

### 2.4 Cell transfection

Sheep GCs were seeded into a six-well dish (5 × 10^5^ cellsperwell ). After reaching 50 % confluency, the GCs were transfected with miR-383 mimics, miR-383 inhibitors, and their negative controls (NCs) (Sangon, Shanghai, China) using Lipofectamine 2000 Transfection Reagent (Invitrogen, Carlsbad, USA). After 2 d , the cells were collected, and the reverse transcription quantitative polymerase chain reaction (RT-qPCR) was used to measure the transfection efficacy. The miRNA sequences are listed in Table 1.

**Table 1:** RNA oligonucleotide sequences used in this study.

Fragment name	Sequence (5^′^–3^′^)
miR-383 mimics	AGAUCAGAAGGUGAUUGUGGCU
miR-383 mimics NC	UUGUACUACACAAAAGUACUG
miR-383 inhibitor	AGCCACAAUCACCUUCUGAUCU
miR-383 inhibitor NC	CAGUACUUUUGUGUAGUACAA

### 2.5 Determination of apoptosis and cell cycle

Annexin V-FITC–PI (V-fluorescein isothiocyanate–propidium iodide) staining (Beyotime, Shanghai, China) was used to identify apoptotic cells. Following transfection, the cells were collected via centrifugation and rinsed with ice-cold PBS. This was followed by treatment with PI (50 $\mathrm{mg}\;{\mathrm{mL}}^{-1}$ ) and Annexin V-FITC (20 $\mathrm{mg}\;{\mathrm{mL}}^{-1}$ ). Flow cytometry was performed to assess the cell distribution. The data were analysed using FlowJo v10 software.

The GCs were seeded into 12-well plates and transfected upon reaching a density of approximately 60 %. Following a 36 h transfection period, the cells were collected in 1.5 mL EP tubes. Then, 1 mL of pre-chilled 70 % ethanol was added to each tube, and the cells were fixed for 24 h at 4 °C . Finally, each cell sample was stained with 500 µL of propidium iodide (50 $µg\;{\mathrm{mL}}^{-1}$ , TianGen, Beijing, China) and incubated for 30 min at 37 °C under dark conditions. A FACSAria SORP flow cytometer (BD Inc., Franklin, NJ, USA) was used to evaluate the cells. FlowJo v10 software (Becton, Dickinson and Company, New York, NY, USA) was used to analyse the data.

### 2.6 EdU cell proliferation assay

Upon reaching 40 %–60 % confluency, the GCs were transfected in the cell culture plates. After 36 h , the cells were treated in accordance with the Cell-Light EdU Apollo567 (5-ethynyl-2'-deoxyuridine) (RiboBio, Guangzhou, China) procedure. An inverted fluorescence microscope (DMi8, Leica, Wetzlar, Germany) was used to randomly capture three images. Image-Pro Plus 6.0 software (Media Cybernetics, Rockville, MD, USA) was used to measure the total number of cells and the number of cells that were EdU positive.

**Table 2:** Primers used in this study.

Gene	Primer sequence (5^′^–3^′^)	Product size (bp)	Tm ( °C )	Reference in GenBank
miR-383	AGAUCAGAAGGUGAUUGUGGCU	–	61	MIMAT0009309
*PCNA*	F: GCGTCTCAGGCGTTCGTAATC	100	61	XM_004014340.5
	R: TGACACGGCTACAGGTACAAGG			
*CCND1*	F: GAGAAACCGCAATAGCGACCT	256	62	XM_027959928.2
	R: GCCCTCCACACACCACAGAA			
*CCND2*	F: GATTGAGGTGGTGCTGCTGAAC	100	62	NM_001127290.1
	R: CGTAGGGGTGCTGGCTTGG			
*BAX*	F: AGAAGCTGAGCGAGTGTCTGAAG	81	63	XM_027978594.3
	R: CCACGGCTGCGATCATCCTC			
Caspase-3	F: GGCTCTGAGTGTTTGGGGAA	131	60	XM_060406952.1
	R: CCTGGACAAAGTTCCGTGGT			
*Bcl-2*	F: CTTCTTTGAGTTCGGAGGGGTCATG	99	63	XM_060405321.1
	R: TTCAGGTACTCGGTCATCCACAGG			
*U6*	F: AGCCTTCAAATCACTGGCTACA	197	60	XM_042252039.2
	R: AGTACCTGCTTACCCATACCT			
*GAPDH*	F: GGTTGTCTCCTGCGACTTCA	216	61	XM_060411595.1
	R: CTCTCTCTTCCTCTCGTGCTCC			

### 2.7 RNA extraction and RT-qPCR

A TRIzol Kit (Biosharp, Hefei, China) was used to extract the total RNA from the GCs. The integrity and concentration of the extracted RNA were assessed using NanoDrop 2000 (Thermo Fisher, MA, USA). The extracted RNA was used for RT-PCR using HiScript II 1st Strand cDNA Synthesis Kit (Vazyme, Nanjing, China). The cDNA synthesis system is described in the supplementary material and Table S1 in the Supplement. RT-qPCR was performed using the ChamQ Blue Universal SYBR qPCR Master Mix (Vazyme, Nanjing, China). The procedures for RT-qPCR are listed in Table S2 in the Supplement. U6 and GAPDH were used as internal reference genes. The primer sequences are listed in Table 2.

### 2.8 Western blot analysis

A radioimmunoprecipitation assay buffer (ServiceBio, Wuhan, China) was used to isolate the total protein from the GCs. Following protein quantitation with a bicinchoninic acid assay kit (Beyotime, Shanghai, China), polyacrylamide gel electrophoresis was performed using 20 µg of protein from the knockout and overexpression groups. Following transfer, the membrane was blocked with 5 % skimmed milk for 2 h . After sealing, the membrane was washed with 1 × tris-buffered saline with 0.1 % Tween^®^ 20 detergent (TBST) for 10 min and subjected to three subsequent washes. Next, the membrane was incubated with a primary antibody at 4 °C overnight and washed thrice with TBST for 10 min . Subsequently, the membrane was incubated for 1 h with a secondary antibody. An enhanced chemiluminescent (ECL) kit (ServiceBio, Wuhan, China) and ECL luminous instrument (Thermo Fisher, MA, USA) were used to develop and visualise the bands.

### 2.9 Luciferase reporter assay

MiRDB (https://www.mirdb.org, last access: 25 October 2024) (Chen and Wang, 2020), miRWalk (http://mirwalk.umm.uni-heidelberg.de, last access: 25 October 2024) (Sticht et al., 2018), and TargetScanHuman 8.0 (https://www.targetscan.org/vert_80/, last access: 25 October 2024) (McGeary et al., 2019) were used to identify putative miR-383-related target genes. miRanda v3.3 predicted a binding site for miR-383 on Bcl-2 based on a free energy calculation. Bcl-2 wild-type and mutant recombinant plasmids were constructed using the pmiRGLO plasmid. The miR-383 mimics, mimics NC, wild-type recombinant plasmid, and mutant recombinant plasmid were co-transfected into 293T cells. The dual-luciferase assay system was used to quantitate the luciferase activity following a 36 h incubation period. The luciferase activity of the sea kidney was considered the control.

### 2.10 Statistical analysis

GraphPad Prism 9.5 software (San Diego, CA, USA) was used to analyse the experimental data based on a two-tailed Student t test. A p value of less than 0.05 was considered statistically significant. The results are presented as the mean ± standard error of the mean.

**Figure 1:**
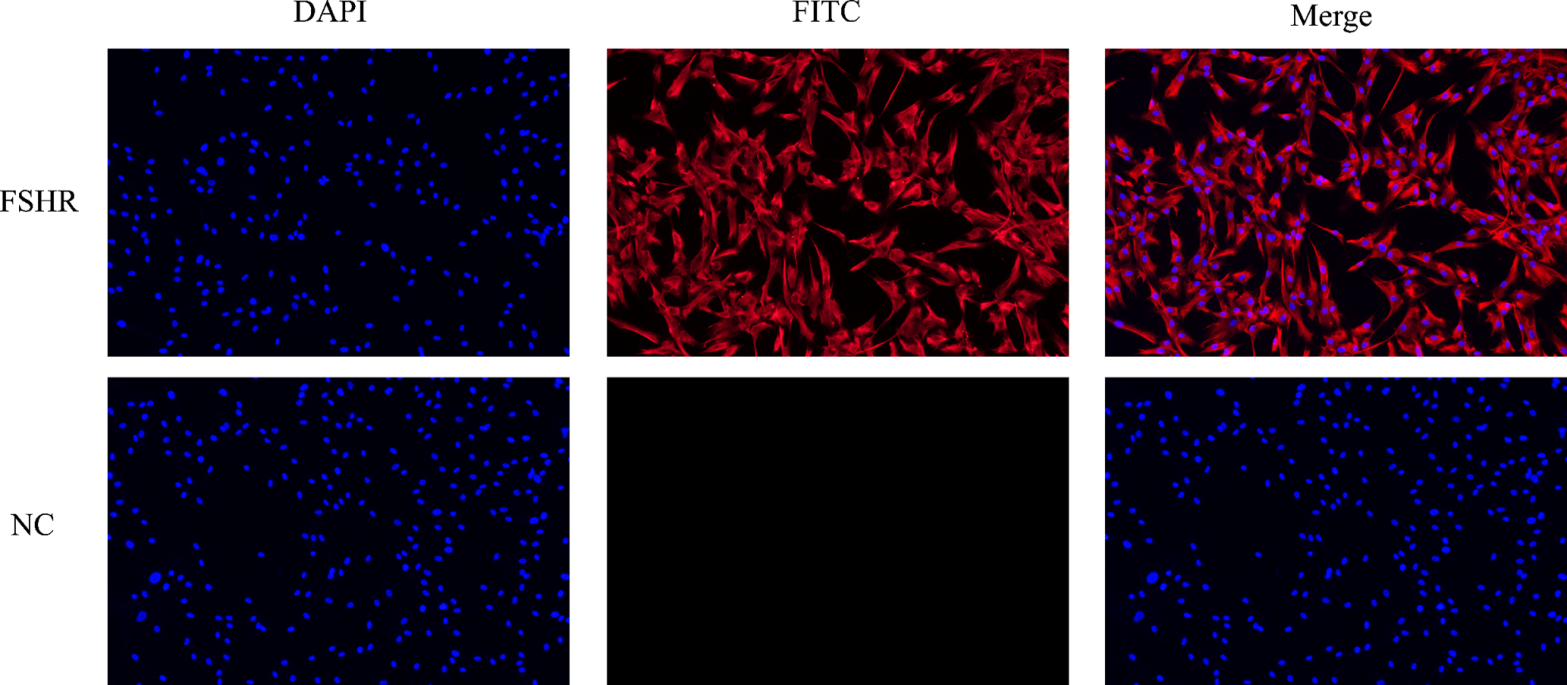
Distribution of the sheep ovarian granulosa cell marker protein FSHR in GCs. Blue: DAPI; red: FSHR; magnification = 200 × ; scale = 50 µm .

## 3 Results

### 3.1 Cell identification

To confirm that the cultured cells were sheep GCs, immunofluorescence was used to detect FSHR expression in the isolated cells. The results demonstrated that FSHR was highly expressed, indicating that the isolated cells were sheep GCs (Fig. 1).

**Figure 2:**
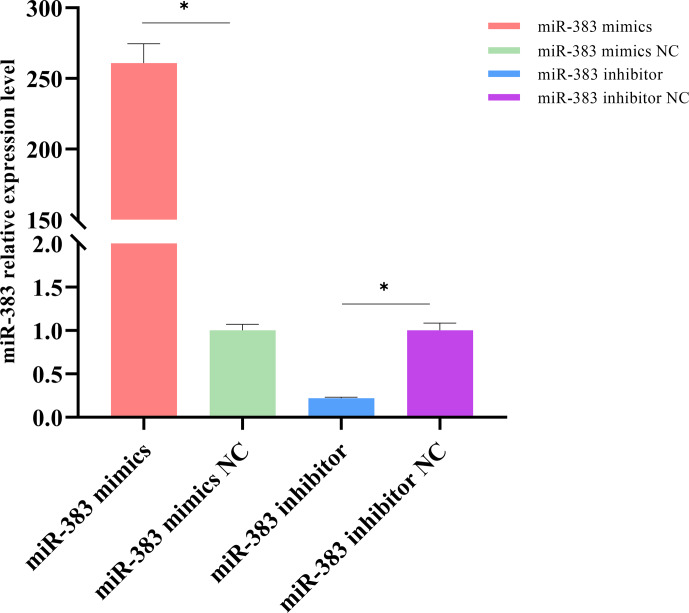
miR-383 expression after transfection with miR-383 mimics and inhibitors in sheep GCs (^∗^ p < 0.05).

### 3.2 Transfection efficiency

To determine the regulatory role of miR-383, miR-383 simulants (miR-383 mimics and mimics NC) and inhibitors (miR-383 inhibitor and inhibitor NC) were transfected into GCs. RT-qPCR was used to measure transfection efficiency (Fig. 2). The results indicated a marked increase in miR-383 expression in the mimics group compared with that in the mimics NC group. In contrast, miR-383 expression considerably decreased in the inhibitor group.

**Figure 3:**
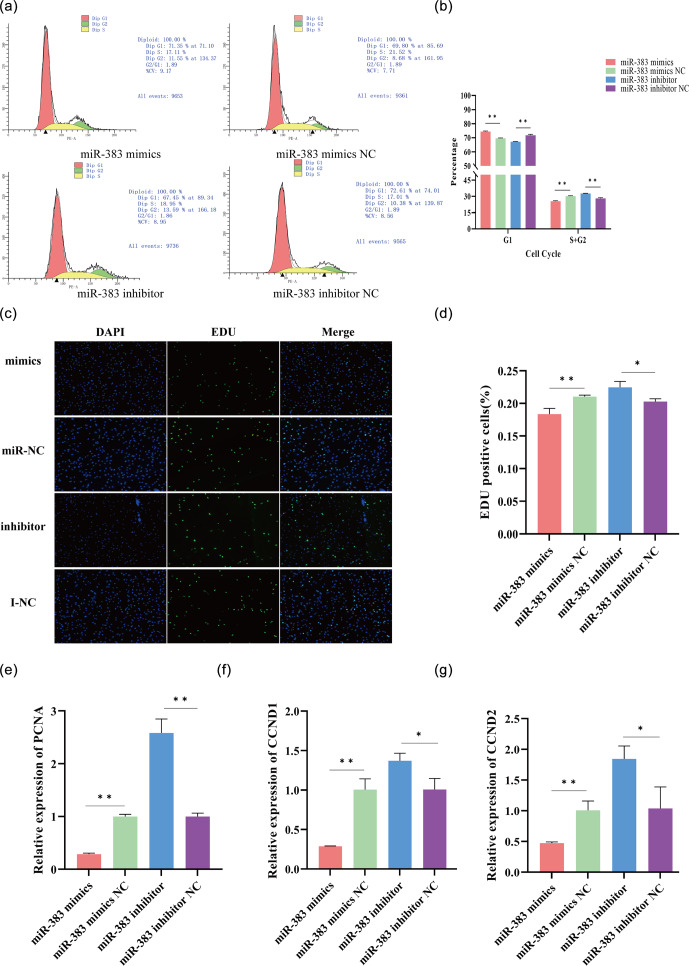
Effect of miR-383 on sheep GC proliferation. **(a, b)** Following transfection with miRNA-383 mimics and inhibitors, flow cytometry showed changes in the cell cycle. **(c, d)** Inverted-fluorescence microscopy images of cell proliferation after transfection with miRNA-383 mimics and inhibitors along with statistical results for the images. **(e–g)** Relative expression levels of *PCNA*, *CCND1*, and *CCND2* mRNA in GCs after transfection with miRNA-383 mimics and inhibitors (^∗^ p < 0.05, ^∗∗^ p < 0.01).

### 3.3 miR-383 suppresses GC proliferation

The cell cycle distribution was examined using flow cytometry to determine whether miR-383 affects cell cycle progression in GCs. Following transfection with miR-383 mimics, the proportion of GCs in the G1 phase dramatically increased, whereas that in the S + G2 phase considerably decreased. Conversely, miR-383 downregulation had the opposite effect (Fig. 3a and b). Next, we used EdU to analyse cell viability. The results indicated that miR-383 overexpression decreased EdU positivity (Fig. 3c and d). In contrast, the viability of GCs in the inhibitor group was significantly higher than that in the inhibitor NC group (Fig. 3c and d). To understand the mechanism by which miR-383 contributes to the growth of sheep GCs, we first measured the relative expression of *PCNA*, *CCND1*, and *CCND2* in the GCs. RT-qPCR indicated that transfection with miR-383 mimics significantly reduced the expression levels of *PCNA*, *CCND1*, and *CCND2* in the GCs, whereas miR-383 downregulation markedly increased the expression of these genes (Fig. 3e–g). Taken together, miR-383 suppresses the growth of sheep GCs.

**Figure 4:**
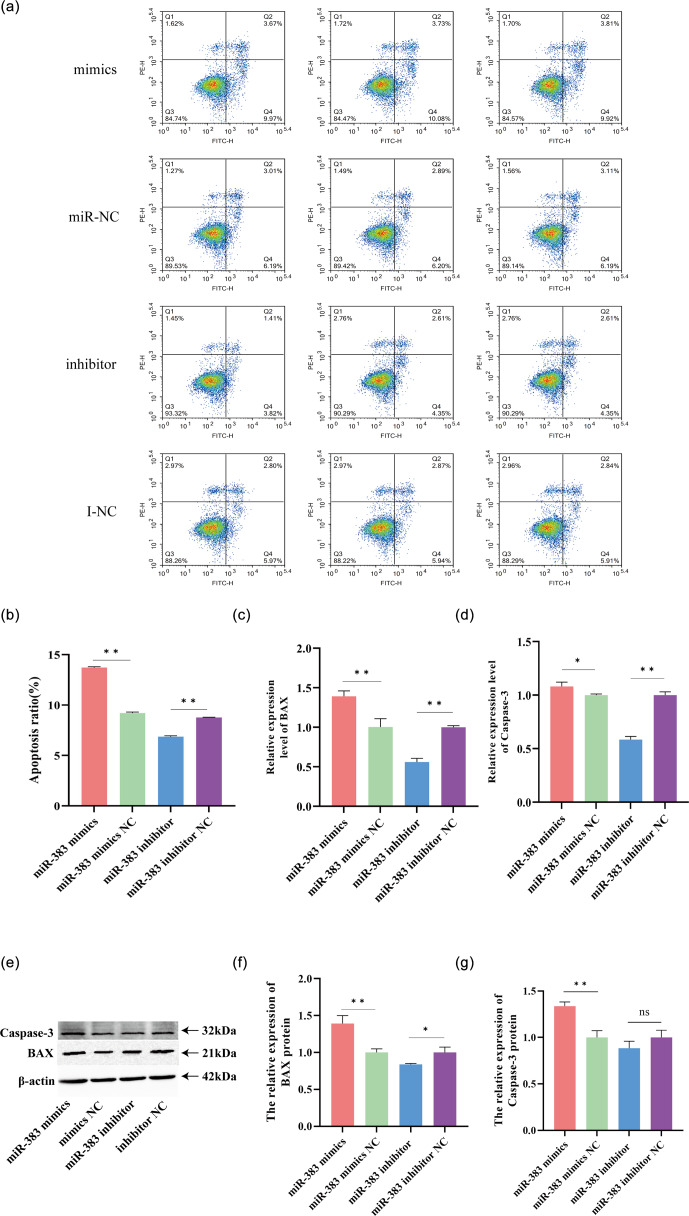
Apoptosis in miR-383-transfected GCs in sheep. **(a, b)** Apoptosis was assessed via flow cytometry. **(c, d)** Expression of the apoptosis marker genes, *BAX* and caspase-3, following transfection with a miR-383 mimics inhibitor and negative controls. **(e)** Protein expression levels of caspase-3, *BAX*, and β -actin. **(f)** The relative expression of the *BAX* protein. **(g)** The relative expression of the caspase-3 protein. (^∗^ p < 0.05, ^∗∗^ p < 0.01; ns: no significance).

### 3.4 miR-383 promotes GC apoptosis

We transfected sheep GCs with miR-383 mimics, miR-383 mimics NC, miR-383 inhibitor and miR-383 inhibitor NC to determine the role of miR-383 in apoptosis using flow cytometry, RT-qPCR, and western blot analysis. Compared with the miR-383 mimics NC group, GCs transfected with miR-383 mimics showed considerably greater rates of early and late apoptosis based on flow cytometry. In contrast, the GCs in the miR-383 inhibitor group exhibited markedly reduced early and late apoptosis rates compared with those in the inhibitor NC group (Fig. 4a and b). RT-qPCR revealed that the expression levels of apoptosis-related *BAX* and caspase-3 genes significantly increased following transfection with miR-383 mimics (Fig. 4c and d). Conversely, a significant decrease was observed in the expression levels of *BAX* and caspase-3 in the inhibitor group (Fig. 4c and d). By assessing the apoptosis marker proteins *BAX* and caspase-3, the regulatory role of miR-383 was further substantiated. The relative expression levels of *BAX* and caspase-3 protein in GCs significantly increased after transfection with miR-383 mimics, whereas the relative expression levels of the apoptosis protein *BAX* in GCs were significantly decreased after transfection with the miR-383 inhibitor (Fig. 4e–g). The results indicate that miR-383 induces GC apoptosis.

**Figure 5:**
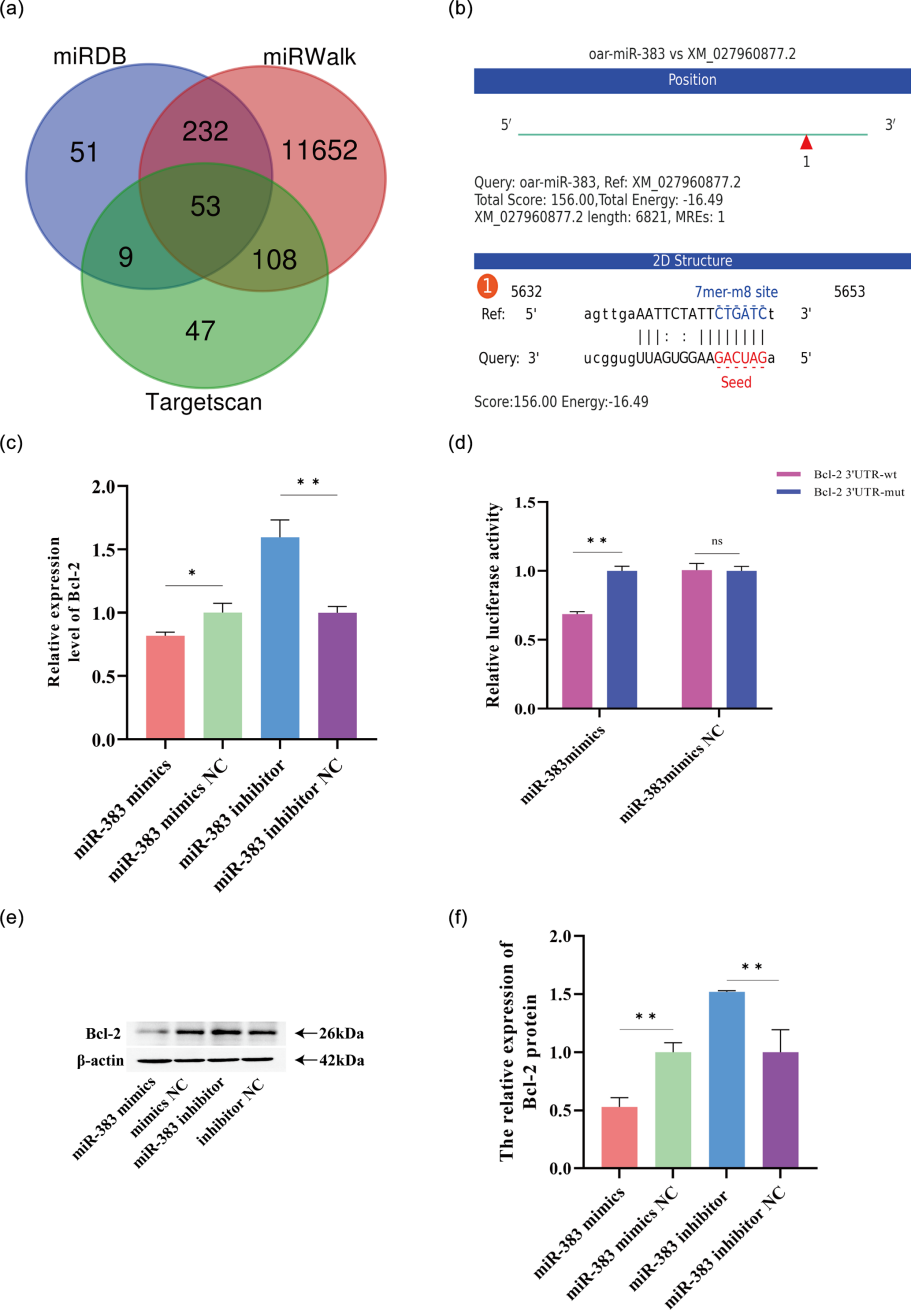
*Bcl-2* is a target gene of miR-383 in sheep GCs. **(a)** Venn diagram of the target gene predicted by miRDB, TargetScan, and miRWalk ( p adj < 0.05, ${\mathrm{log}}_{2}$ fold change < 0). **(b)** Results of the binding energy prediction by miRanda. **(c)** Relative expression of the miR-383 target gene *Bcl-2* after transfection with the miR-383 mimic inhibitor and negative controls. **(d)** Target relationship between miR-383 and *Bcl-2* verified using the Dual-Luciferase Reporting Assay System. **(e)** Protein expression of *Bcl-2*. β -Actin was used as an internal reference protein. **(f)** The relative expression of the *Bcl-2* protein. (^∗^ p < 0.05, ^∗∗^ p < 0.01; ns: no significance).

### 3.5 Bcl-2 serves as a target gene for miR-383

miRNA-383 is essential for controlling the proliferation and apoptosis of GCs. Next, we used TargetScanHuman8.0, miRDB, and miRanda to identify *Bcl-2* as a potential target gene (Fig. 5a and b). *Bcl-2* expression was measured following miR-383 overexpression and inhibition to determine the relationship exists between the two molecules. RT-qPCR revealed that *Bcl-2* expression following transfection with the miR-383 was significantly lower than that in the mimic NC group. Transfection with miR-383 inhibitor showed the opposite effect (Fig. 5c). The results of the western blot analysis were consistent with those of RT-qPCR (Fig. 5e and f). A dual-luciferase reporter assay was used to detect the miR-383 binding site. Compared with the miR-383 mimics and pmiRGLO-*Bcl-2*–3^′^UTR-WT co-transfection group, the luciferase activity was significantly lower in the miR-383 mimics and pmiRGLO-*Bcl-2*-3^′^UTR-MUT groups (Fig. 5d). The luciferase activity of the miR-383 mimics NC and pmiRGLP-*Bcl-2*-3^′^UTR co-transfected groups was not significantly different from that of miR-383 mimics NC and pmiRGLO-*Bcl-2*-3^′^UTR-MUT co-transfected groups (Fig. 5d). The results indicated that *Bcl-2* is a target gene of miR-383.

## 4 Discussion

GCs, an important somatic cell type in ovaries, play a regulatory role in follicular development, differentiation, ovulation, and atresia (Zhu et al., 2019; Chen et al., 2024). Over the past few years, studies have identified numerous miRNAs associated with mammalian follicular development and atresia through high-throughput sequencing, knockdown of key genes during miRNA production, and overexpression or inhibition of miRNAs (Zhang et al., 2019; Donadeu et al., 2017). Among them, miR-383 belongs to the differentially expressed miRNA obtained by high-throughput sequencing of different developing follicles (Na et al., 2022; Salilew-Wondim et al., 2014). miRNAs play a regulatory role in GC apoptosis through the inhibition or degradation of their target genes by binding to their 3^′^UTR (Jiajie et al., 2017). In sheep ovarian GCs, miR-1306 promotes GC apoptosis by targeting *BMPR1B* (Abdurahman et al., 2022). miR-23a-3p overexpression inhibits *HMGA2* expression in human ovarian GCs, suppresses cell proliferation, and promotes apoptosis (Wei et al., 2023). In chicken GCs, miR-34a-5p overexpression can induce autophagy and cell death by targeting *LEF1*, thereby mediating to mediate the HPO-YAP signalling pathway (Han et al., 2023).

Studies have shown that miR-383 regulates cell proliferation by regulating the genes associated with the cell cycle. During spermatogenesis, miR-383 inhibits cyclin D1 expression through direct targeting, thereby blocking spermatogonial cells in the G1 phase as well as spermiogenesis (Tian et al., 2013). In this study, miR-383 overexpression in sheep GCs, the expression of the cell-cycle-related genes *PCNA*, *CCND1*, and *CCND2*, arrested the cell cycle at the G1 phase and inhibited cell proliferation. The inhibitory effect of miR-383 on cell growth may be reversed by suppressing miR-383 expression. Our results indicate that miR-383 can regulate the proliferation of ovarian GCs by inhibiting *PCNA* and *CCND1*/*CCND2* expressions.

*BAX* and caspase-3 are key regulatory genes that control apoptosis (Cregan et al., 1999). Upon receiving apoptotic signals, *BAX* is activated, which leads to the disruption of mitochondrial membrane integrity, release of cytochrome c, and cleavage and activation of caspase-3, thereby promoting cell apoptosis (Juin et al., 2002). Studies have shown that miR-383, a type of miRNA that regulates cell apoptosis, promotes apoptosis by regulating the expression of *BAX* and caspase-3. miR-383 overexpression increases the expression levels of *BAX* and caspase-3 to induce apoptosis in breast cancer cells (Azarbarzin et al., 2021). Based on these findings, we used flow cytometry to verify that miR-383 overexpression promotes granulocyte apoptosis, whereas the opposite effect was observed upon the inhibition of miR-383 expression. RT-qPCR and western blot analysis revealed that miR-383 overexpression increases the expression levels of the pro-apoptotic genes *BAX* and caspase-3, partially explaining the role of miR-383 in promoting granulocyte apoptosis.

The *Bcl-2* gene, which is the first member of the *Bcl-2* family to be implicated in apoptosis, acts as a main switch along with *BAX* to regulate apoptosis (Adams and Cory, 2007). *Bcl-2* is typically located within the mitochondria or endoplasmic reticulum, where it can impede the apoptotic process by obstructing the binding of BH3-only proteins to their activators and sensitisers. Additionally, it interacts with inositol trisphosphate receptors and membrane glycoprotein complexes, acting as a membrane calcium channel on the endoplasmic reticulum to inhibit calcium-mediated apoptosis (Shamas-Din et al., 2013; Greenberg et al., 2014). Studies have shown that miRNA can target *Bcl-2* and regulate apoptosis. During the progression of rheumatic heart disease, miR-1183 can directly target *Bcl-2* to inhibit its expression, thereby aggravating the disease (Li et al., 2020). In breast cancer cells, miR-148a can suppress tumour growth and promote apoptosis through binding with to 3^′^UTR of *Bcl-2* (Li et al., 2017). In rat hepatocytes, miR-383-5p targets *Bcl-2* to inhibit its translation and induce stem cell apoptosis (Xu et al., 2020), which is consistent with our findings. In our study, RT-qPCR, western blot analysis, and dual-luciferase experiments demonstrated that miR-383 can regulate ovine GC apoptosis by targeting the *Bcl-2* gene.

Since Rosalind C. Lee et al. (Lee et al., 1993) first discovered miRNA, many researchers have shown interest in this field. Studies on the reproductive physiology of female mammals have shown that miRNA is a key factor in regulating ovarian reproductive physiology, including GC proliferation, apoptosis, and endocrine function. We demonstrated that miR-383 inhibits GC proliferation by targeting *Bcl-2* and promotes GC apoptosis. These findings provide a foundation for further studies on the role of miR-383 in GCs.

## 5 Conclusions

The miR-383 overexpression suppresses ovine GC proliferation and induces apoptosis by targeting *Bcl-2*. These findings highlight the regulatory role of miR-383 in sheep GCs and provide a theoretical foundation for developing strategies to modulate female mammalian reproduction using miRNAs.

## Supplement

10.5194/aab-68-287-2025-supplement The supplement related to this article is available online at https://doi.org/10.5194/aab-68-287-2025-supplement.

## Data Availability

The data are available from the corresponding author upon request.
