# Supplementary material for: miR-383 regulates sheep granular cell proliferation and apoptosis by targeting Bcl-2
Source: Arch Anim Breed. 2025 May 15;68(2):287–97. doi: 10.5194/aab-68-287-2025 (PMC13282089; doi:10.5194/aab-68-287-2025)
Supplement: The supplement related to this article is available online at https://doi.org/10.5194/aab-68-287-2025-supplement. [file aab-68-287-2025-supplement.zip › aab-68-287-2025-supplement-title-page.pdf]

Supplement of Arch. Anim. Breed., 68, 287–297, 2025
https://doi.org/10.5194/aab-68-287-2025-supplement
© Author(s) 2025. CC BY 4.0 License.

Open Access
Archives Animal Breeding

Supplement of

miR-383 regulates sheep granular cell proliferation and apoptosis by targeting *Bcl-2*

Binglei Zhang et al.

Correspondence to: Yuqin Wang (wangyq6836@163.com)

- aab-68-287-2025-supplement-title-page.pdf
- Supplemental
  - Table S1 The cDNA synthesis system.xlsx
  - Table S2 The procedures of RT-qPCR.xlsx

The copyright of individual parts of the supplement might differ from the article licence.
